# Postoperative antibiotics and infection rates after implant-based breast reconstruction: A systematic review and meta-analysis

**DOI:** 10.3389/fsurg.2022.926936

**Published:** 2022-08-17

**Authors:** Yang Hu, Xuan Zhou, Xiaofei Tong, Xiangyu Chen, Mingzhu Wang, Xianrui Wu, Peiting Li, Fengjie Tang, Jianda Zhou, Ping Li

**Affiliations:** ^1^ Department of Plastic Surgery of Third Xiangya Hospital, Central South University, Changsha, China; ^2^ Department of Epidemiology and Health Statistics, Xiangya School of Public Health, Central South University, Changsha, China

**Keywords:** meta-analysis, postoperative antibiotics, infection, breast reconstruction, implant-based breast surgery

## Abstract

**Purpose:**

Infection is the most common complication following breast implant surgery. Nevertheless, the systematic administration of antibiotics after breast implant surgery has been subjected to controversial debate. In this study, we sought to elucidate the association between infection and the use of antibiotics as an aftermath of breast implantation surgical procedures.

**Methods:**

Relevant studies were identified from PubMed, Web of Science, and EMBASE search mining. The extracted data included study type, basic characteristics, administrated antibiotic information, and clinical outcomes. Random-effects models were utilized to estimate outcomes, while study quality, statistical bias, and heterogeneity were also analyzed.

**Results:**

A total of 7 studies involving a total of 9,147 subjects were included. The results demonstrated that the use of antibiotics after breast implantation reduced the incidence of infection (risk ratio [RR]: 0.65, 95% CI, 0.46–0.90). Nevertheless, smoking, obesity and diabetes type II are risk factors for postoperative infections. Sensitivity analysis verified the robustness of the results.

**Conclusions:**

Our study identified the administration of antibiotics after breast implantation as an intervention that decreased the incidence of infection. Smoking, obesity, and diabetes type II are risk factors for postoperative infections. These findings strongly suggest that timely and effective antibiotic interventions will be crucial in future clinical practice, which may reduce the risk of postoperative infection following breast implantation.

## Introduction

Breast reconstruction surgery approaches after breast cancer and/or mastectomy can provide physiological and psychological comfort to breast patients by surgically restoring the shape of the breast (1). To this extent, an increasing case of patients are opting for breast reconstruction after breast cancer surgery, which robustly magnifies the clinical importance of breast reconstruction. Implant-based breast reconstruction is the most popular option, accounting for approximately 80% of post-operative breast cancer reconstructions (2, 3). Despite the growing popularity of the approach, infection is a major complication after breast implant surgery. In particular, breast reconstruction after mastectomy and cancer radiotherapy is associated with a higher risk of infection (4).

Previous study showed that the infection rate associated with breast implant surgery can be as high as 35.40% (5). Nonetheless, according to the Centers for Disease Control (CDC) guidelines, breast surgery is considered as an aseptic-field procedure and therefore a maximum of 24 h of perioperative antibiotics is currently recommended (6). In fact, breasts are not sterile. What's more, endogenous skin flora colonizing the nipple can spread to deeper breast tissue through the milk ducts or during surgical procedures, which can lead to infection. Coagulase-negative staphylococci can be isolated from the breast in more than half of women undergoing breast augmentation or breast reduction. Other skin flora frequently isolated from the breast including *diphtheria-like*, *Lactobacillus*, *Bacillus*, *beta-hemolytic streptococci*, and *Propionibacterium acnes* (7). In particular, the incidence of methicillin-resistant *Staphylococcus aureus* (MRSA) infections is increasing (8, 9). Other common Gram-positive bacteria including *Streptococcus*, although recent reports showed an increased incidence of Gram-negative bacterial infections, such as *Pseudomonas* (10). Therefore, in the long term, bacteriological treatment with prophylactic antibiotics in patients with breast implants is necessary (11, 12).

The American Society of Plastic Surgery has developed guidelines for implant reconstruction, but in terms of infection prevention, they only cover the use of perioperative antibiotics and do not address the use of postoperative antibiotics. However, in a survey of members of the American Society of Plastic Surgeons (ASPS), roughly 72 percent of physicians prefer to continue using antibiotics following breast reconstruction surgery for a period of time, usually longer than 24 h (13). In the following years after this milestone study, several physicians have tried a plethora of therapeutic approaches, without achieving universal guidelines and concordance. On the contrary, other studies demonstrated that postoperative antibiotics failed to reduce the incidence of infection (14–16), while Townley et al. suggested that only preoperative antibiotics and postoperative antibiotics were equally effective on reducing the infection rates (17). Nevertheless, some cohort prospective studies have indicated that postoperative antibiotic use was effective in reducing the incidence of infection (18–21). Meanwhile, a previous meta-analysis on the use of extended prophylactic antibiotics in breast reconstruction which only focused on immediate breast reconstruction, fail to reach on a definite conclusion on the causal relationship between antibiotic use and infection after breast implantation (22). Therefore, antibiotics usage after breast implantation remains highly controversial, which introduces a significant clinical dilemma regarding the risk of infection and antibiotic use after breast implantation.

The purpose of this article is to elucidate the effectiveness and feasibility of postoperative antibiotics on controlling the incidence of infection after breast implantation, in an effort to optimize the clinical decisions for the management of these patients.

## Materials and methods

### Search strategy

**Figure 1:**
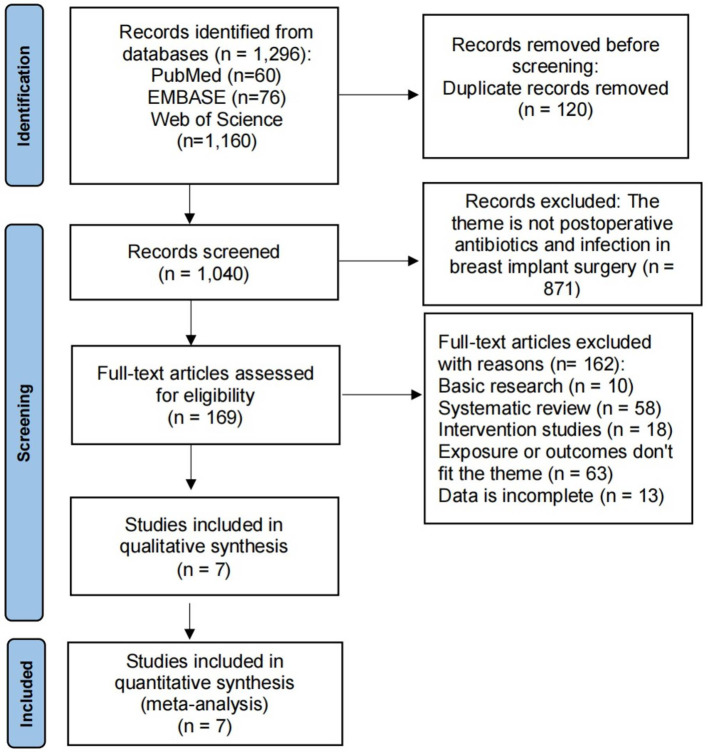
PRISMA flow diagram.

This systematic review and meta-analysis were conducted according to the Preferred Reporting Items for Systematic Reviews and Meta-Analyses (PRISMA) guidelines. A systematic research strategy was constructed based on the following question, whether the postoperative antibiotic usage can reduce the incidence of infectious outcomes in breast implant surgery. To address this question, two authors independently identified studies published in English as of September 30th, 2021, through the following online databases: PubMed, Embase, and Web of Science. Search terms were used in different combinations to identify the maximum number of relevant studies in these databases. Where possible, subject headings, web banners, and other forms of indexing were utilized. The detailed search strategy used in this study is outlined in Figure 1. In addition, we collected relevant articles that were not included in the search but satisfied the search requirements.

### Exposure and outcomes

In our study, exposure is the administration of antibiotics after breast implantation. Specifically, we defined the postoperative antibiotics uniformly as the use of antibiotics after the operation, usually for no more than 24 h. The outcome is the occurrence of topical infection, which was defined by the CDC guidelines as septic drainage, positive bacterial cultures, erythema around the incision, surgical cleaning of the surgical incision site, and physician diagnosis.

### Inclusion and exclusion criteria

While screening articles titles and abstracts, we intentionally expanded the inclusion criteria to identify any relevant studies. As a first inclusion criterion, studies were included if they reported an association between antibiotics and breast implant surgical infection and were published in English. Secondly, the full text of the selected studies was reviewed. Studies were included if they met the following study design criteria: the design was observational; the study population had at least two groups (one with and one without postoperative antibiotic use); or demonstrated sufficient information to allow accurate risk estimation, subsequent statistical analyses and calculation of the corresponding 95% CI. Studies were excluded if they were review articles, case reports, letters to the editor, or conference abstracts, were not controlled, provided unclear or incomplete data or were redundant publications. In the case one of a study from the same population, only the most comprehensive or most recently published study was included.

### Data abstraction

Using self-designed tables, two researchers independently extracted the following data and demographics from each study: first author, year of publication, geographic region, study design, type of reconstruction, antibiotic application regimen, duration of postoperative antibiotic, follow-up time, infection rate, route of administration, time to infection diagnosis, sample size, quality score, control confounders and risk estimates, and the corresponding 95% CIs (adjusted CIs was extracted, if available). Any discrepancies were handled through conversation or using an independent third reviewer, to increase the methodology soundness of the study.

### Quality assessment

Two researchers (Y.H. and X.Z.) independently assessed the methodological quality of studies using the Newcastle-Ottawa Scale (NOS) for quality evaluation of cohort studies. There were eight items in the NOS. Each study received an objective rating of 1–9 stars based on three distinct criteria: selection, comparability, and result (cohort studies). A final median score of 6 and above was considered as excellent.

### Statistical analysis

Risk ratio (RR) was used as a reference value to measure the association between the use of antibiotics after breast implant surgery and infection. Random effects model meta-analysis was used as a tool to calculate combined RRs and 95% CIs (23). Heterogeneity was assessed by the Chi-square test for categorical variables (*p* < 0.05 represented statistically significant heterogeneity) and *I*^2^ statistic (*I*^2^ > 75% indicated an extremely high level of heterogeneity, 51%–75% showed a high level of heterogeneity, 26%–50% demonstrated a moderate-level of heterogeneity, and ≤25% signified a low level of heterogeneity) (24, 25). The Chi-square test was used to estimate whether the variance between studies was ascribed to chance and the *I*^2^ statistic was used to estimate the proportion of total variation in prevalence evaluations owing to statistical heterogeneity instead of sampling error. Publication bias of included articles was assessed by using the Egger test (*p* < 0.05 indicated statistically significant differences). Subgroup analysis included geographic region (e.g., Canada, USA), duration of postoperative antibiotics use (e.g., >48 h, until drainage removal or unknown), confounders controlled (e.g., none of the confounder controlled, one or more confounders controlled), sample size (e.g., ≤500, >500) publication years (e.g., before 2018, 2018 or after) and quality assessment (e.g., ≤7, >7). To assess the robustness of the meta-analysis results, sensitivity analyses were performed, which involved performing meta-analyses after eliminating one included research at a time and comparing the results before and after removal. RevMan version 5.4.1 (The Nordic Cochrane Centre, Cochrane Collaboration, Copenhagen, Denmark) and R version 4.0.5 were used for all statistical analyses (The R Foundation for Statistical Computing).

## Results

### Features of the selected studies

A total of 1,296 articles were identified by system search and manual search. Among them, 1,040 unique articles were included after the exclusion of duplicate publications, 169 articles were retained after title and abstract screening, falling to 82 articles that were included following full-text screening. After the inclusion criteria procedure, 7 articles were ultimately included in the meta-analysis (see Figure 1) (14–20). These articles were all published between 2012 and 2021, and all these articles were cohort studies, involving a total of 9,147 subjects. Details of the included articles are shown in Table 1.

**Table 1:** Selected characteristic of 7 cohort studies.

Reference	Study period	Geographic region	Type of reconstruction	Sample size	Follow-up (months)	Experimental group antibiotics	Experimental group infection rate	Control group antibiotics	Control group infection rate	Observation time from surgery to infection (days)	Duration of postoperative antibiotics	Quality score
Feras Yamin 2021	2005–2018	USA	Expander implantation	529	5	Perioperative antibiotics + Postoperative antibiotics	62/288 (21.53%)	Perioperative antibiotics	56/241 (23.24%)	NA	Experimental group: >48 h Control grou*p*:<48 h	7
Michael Holland 2021	2015–2018	USA	Expander implantation	221	Average 18	Perioperative antibiotics + Postoperative antibiotics	8/115 (7%)	Perioperative antibiotics	26/106 (24.5%)	NA	Experimental group: until drainage removal (>48 h) Control grou*p*:<48 h	8
Kavitha Ranganathan 2018	2010–2014	USA	Expander implantation + Prosthesis implantation	7,443	At least 6	Postoperative antibiotics	413/6049 (6.83%)	Preoperative antibiotics	105/1394 (7.53%)	Within 180 days	NA	9
Meghan C. McCullough 2016	2005–2011	USA	Expander implantation	378	NA	Preoperative antibiotics + Postoperative antibiotics	24/200 (12.0%)	Preoperative antibiotics	24/178 (13.5%)	Median time: 29 days	NA	7
William A. Townley 2015	2008–2012	Canada	Prosthesis implantation	188	14.9 ± 8.8	Perioperative antibiotics + Postoperative antibiotics	9/94 (10%)	Perioperative antibiotics	11/94 (12%)	NA	Experimental group: until drainage removal Control group: perioperative period only	9
Yash J. Avashia 2013	2007–2010	USA	Expander implantation	138	6.5	Preoperative antibiotics + Postoperative antibiotics	8/119 (6.72%)	Preoperative antibiotics	6/19 (31.58%)	Median time: 18 days	Experimental group: >48 h Control grou*p*: <48 h	7
John L. Clayton 2012	2007–2010	USA	Expander implantation	250	NA	Preoperative antibiotics + Postoperative antibiotics	21/116 (18.1%)	Preoperative antibiotics	46/134 (34.3%)	Experimental group: 256 ± 182 Control group: 90 ± 93	Experimental group: until drainage removal Control group: preoperative period only	9

NA, not applicable.

The types of procedures in the McCullough et al. (14) and Ranganathan et al. (15) studies were all immediate breast reconstruction approaches. In addition to this, subjects in three studies involved expander placement, which was performed in both the anterior thoracic and submuscular planes (14, 16, 20). Moreover, five studies addressed the duration of antibiotic application after breast implant reconstruction, including >48 h and until drainage removal (16–20). For the specific application of antibiotics, four of the included studies noted the use of cephalosporin antibiotics for general patients and clindamycin or vancomycin for allergic patients (14, 17–19). In one study, the majority of subjects chose methomyl/sulfamethoxazole. For patients with sulfonamides allergy, doxycycline and clindamycin were used (20). Another study grouped the duration of postoperative antibiotic application as 1–5 days, 1–5 days and >10 days, however, the risk of infection for all three group was not statistically significant compared to patients who did not use postoperative antibiotics (15). The most common bacteria found in bacterial cultures of infected tissues were methicillin-sensitive *Staphylococcus aureus* (MSSA), followed by methicillin-resistant *Staphylococcus aureus* (MRSA) and *Staphylococcus epidermidis* (14, 20).

### Postoperative antibiotics and infection

**Figure 2:**
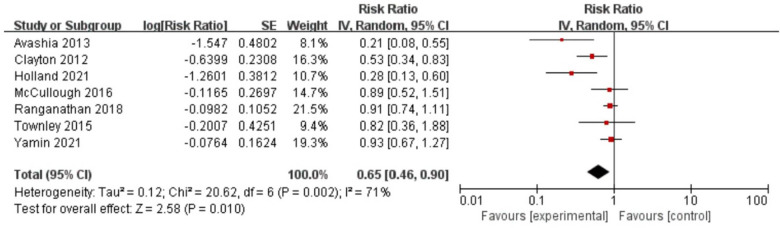
Forest plot for the relationship between antibiotic use and infection after breast implantation.

The included studies reported RRs ranging from 0.21 to 0.93 (see Figure 2). A pooled meta-analysis of these risk estimates yielded an RR of 0.65 (95% CI, 0.46–0.90), with acceptable heterogeneity (*I*^2^ = 71%, *p* < 0.05), which means that postoperative antibiotics is a protective factor for infection after breast implant reconstruction. Notably, the Egger test showed no potential publication bias (*z* = −2.47, *p* = 0.0564). Sensitivity analyses were repeated as outlined in the methods, after excluding each included study in the meta-analyses. The results of the sensitivity analysis showed that excluding any of the studies did not substantially alter the risk estimates for postoperative antibiotic use (RRs between 0.57 and 0.73; see Table 2).

**Table 2:** Sensitivity analysis for the association between antibiotic use and infection after breast implantation.

	RR	95% CI	95% CI	*p*-value	*I*^2^ (%)
Avashia 2013	0.7218	0.5289	0.9850	0.0399	61.5
Clayton 2012	0.6366	0.3980	1.0185	0.0596	70.9
Holland 2021	0.7261	0.5315	0.9920	0.0444	61.9
McCullough 2016	0.5816	0.3679	0.9194	0.0203	75.5
Ranganathan 2018	0.5699	0.3606	0.9007	0.0160	70.8
Townley 2015	0.5982	0.3815	0.9379	0.0251	75.7
Yamin 2021	0.5712	0.3631	0.8986	0.0154	74.2

**Figure 3:**
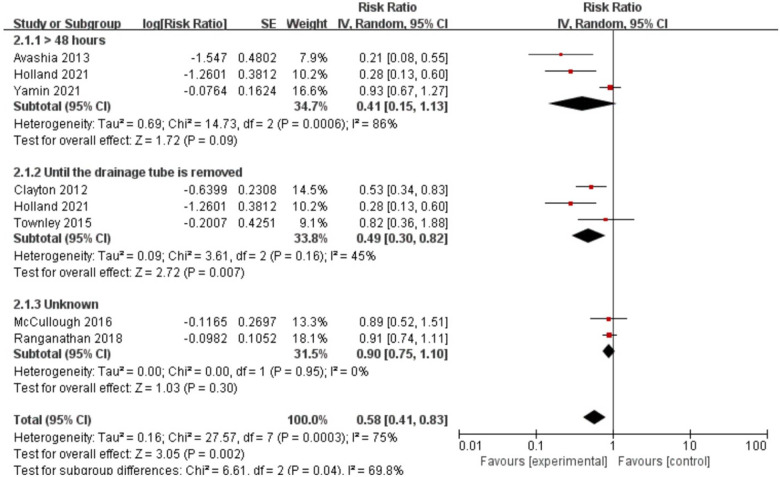
Forest plot for the subgroup of duration of postoperative antibiotic use and infection.

Table 3 showed the results of the subgroup analysis between postoperative antibiotics and infection rates after breast implant surgery. Following subgroup analysis, variables involving geographic region (test for subgroup differences [TSD]: *I*^2^ = 0%), duration of postoperative antibiotics use (TSD: *I*^2^ = 69.80%), sample size (TSD: *I*^2^ = 80.30%), publication years (TSD: *I*^2^ = 0%), quality assessment (TSD: *I*^2^ = 0%) and confounders controlled (TSD: *I*^2^ = 33.30%) were determined as potential heterogeneity moderators. When stratified by duration of postoperative antibiotic use, antibiotic use until drainage removal remains an effective measure to prevent infection after breast reconstruction (RR: 0.49, 95% CI, 0.30–0.82) (see Figure 3).

**Table 3:** Subgroup analyses for the association between antibiotic use and infection after breast implantation.

Subgroup	No. of studies	RR (95% CI)	*I*^2^ (%)	*p*-value for heterogeneity	Test for subgroup differences		
					*χ* ^2^	*p* value	*I*^2^ (%)
Geographic region					0.34	0.56	0
Canada	1	0.82 [0.36, 1.88]	–	–			
USA	6	0.62 [0.43, 0.90]	76	0.001			
Duration of postoperative antibiotic use					6.61	0.04	69.8
>48 h	3	0.41 [0.15, 1.13]	86	<0.001			
Until drainage removal	3	0.49 [0.30, 0.82]	45	0.16			
Unknown	2	0.90 [0.75, 1.10]	0	0.95			
Sample size					5.08	0.02	80.3
>500	2	0.91 [0.77, 1.08]	0	0.91			
≤500	5	0.50 [0.31, 0.82]	64	0.03			
Publication years					0.22	0.64	0
Before 2018	4	0.57 [0.34, 0.96]	61	0.05			
2018 or after	3	0.68 [0.41, 1.12]	84	0.002			
Quality assessment					0.02	0.88	0
≤7	3	0.65 [0.34, 1.25]	77	0.01			
>7	4	0.61 [0.37, 1.00]	75	0.007			
Confounders controlled					1.50	0.22	33.3
None confounders controlled	3	0.41 [0.15, 1.13]	86	<0.001			
One or more confounders controlled	4	0.79 [0.60, 1.03]	35	0.20			

### Risk factors for infection

**Figure 4:**
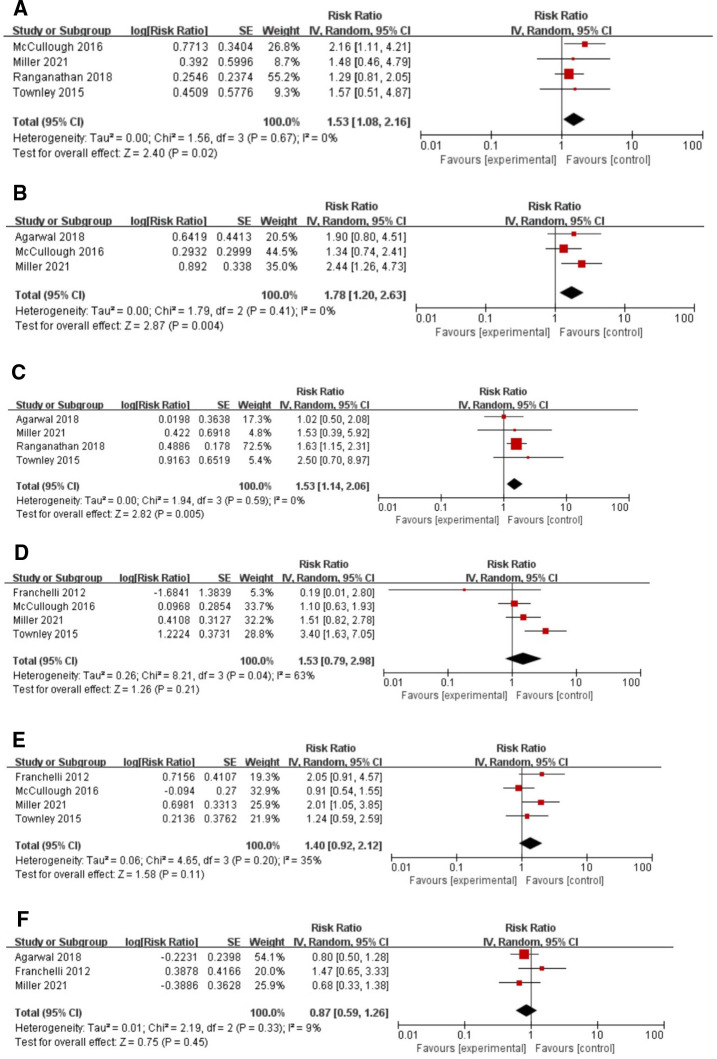
Forest plots for the meta-analyses. (**A**) Smoking; (**B**) Obesity; (**C**) Diabetes; (**D**) Adjuvant radiation; (**E**) Adjuvant chemotherapy; (**F**) Neoadjuvant chemotherapy.

During quantitative analysis, smoking, obesity, and diabetes type II were clearly identified as risk factors for infection after breast implant surgery. Specifically, non-smokers were 1.53 times less likely to have an infection than smokers (RR: 1.53, 95% CI, 1.08–2.16), and with a small heterogeneity (*I*^2^ = 0%, *p* < 0.05) (see Figure 4A). Obesity and postoperative infection analysis showed that the risk of postoperative infection in obese patients was 1.78 times higher than that in non-obese patients (RR: 1.78, 95%CI, 1.20–2.63, *I*^2^ = 0%, *p* < 0.005) (see Figure 4B). In the analysis of diabetes for occurrence of infection, patients with diabetes were 1.53 times more likely to have an infection compared to patients without diabetes (RR: 1.53, 95%CI, 1.14–2.06, *I*^2^ = 0%, *p* = 0.005) (see Figure 4C). However, in the analysis of infection and other associated factors, including adjuvant radiation, adjuvant chemotherapy and neoadjuvant chemotherapy, the RRs were 1.53 (95% CI, 0.79–2.98, *p *> 0.05) (see Figure 4D), 1.40 (95%CI, 0.92–2.12, *p *> 0.05) (see Figure 4E) and 0.87 (95%CI, 0.59–1.26, *p *> 0.05) (see Figure 4F), respectively, while neither of which factors reached statistical significance.

## Discussion

Does postoperative antibiotics application reduce the chance of infection after breast implant reconstruction? Based on the importance of this research topic, a growing number of researchers have investigated the association between antibiotic use and infection after breast implantation in recent years, but with no concordant results. The aim of our study was to elucidate the relationship between antibiotic use and infection after breast implantation.

After vigorous selection criteria and statistical analysis, we demonstrated that the use of antibiotics after breast implantation can reduce the incidence of infection (RR: 0.65, 95% CI, 0.46–0.90). More importantly, our study discovered that antibiotic use until drainage removal was beneficial in lowering infection rates after breast implantation (RR: 0.49, 95% CI, 0.30–0.82). Furthermore, the study quantified smoking, obesity, and diabetes as risk factors for infection during breast implantation. Therefore, we can provide robust evidence to the highly controversial issue of antibiotic utilization after breast implant reconstruction surgery. These results will provide great support to clinicians for the use of antibiotics in their clinical practice.

Infection following breast implant restoration is considered to be caused by a combination of causes. Firstly, numerous studies have shown that infection and implant failure are associated with endogenous skin flora colonizing the nipple, including *Staphylococcus aureus, Streptococcus,* and *Lactobacillus*, which is a considerable bottleneck in the patients' quality of life and an economical burden on the healthcare system (18, 19, 26–29). Secondly, residual cavity after mastectomy was considered as a risk factor for infection. Surgical complications and techniques, such as damage to surrounding blood vessels and removal of lymph nodes may ultimately result in the formation of localized hematomas and poor lymphatic drainage in the breast, leading to bacterial growth and reproduction, increasing the chance of local advanced infection (19). Thirdly, incorrect placement of breast implants can cause excessive tension, which can lead to skin necrosis, fat necrosis, flap necrosis, nipple areola necrosis, or glandular necrosis, as well as infection. In most cases, the infection is caused by local tissue ischemia (30). Additionally, several studies have discovered that utilizing surgical drains raises the risk of breast implant infection by up to fivefold (31). These factors can increase the chances of infection and even aggravate it, causing the emergence of more resistant bacteria, which ultimately affects the efficacy of antibiotic use (32).

Cefazolin is the antibiotic of choice for antibiotic prophylaxis after prosthetic joint and prosthetic cardiovascular material implantation based on the CDC guidelines. Nevertheless, there are no updated recommendations for breast implant surgery (6). Although cefadroxil is the empirical drug for the treatment of infections in breast surgery, some studies have shown a higher failure rate of cefadroxil for skin and soft tissue infections. An additional concern is the increasing resistance of staphylococci to cephalosporin and other *β*-lactam antibiotics (33). As a result, the Association for Breast Surgery (ABS) recommendations for implant reconstruction recommended a single intravenous dose of antibiotics at the time of anesthesia induction, especially in cases where antibiotic use is debatable. Antibiotics that are commonly prescribed include intravenous first- or second-generation cephalosporins. Non-*β*-lactam antibiotics with a sufficiently broad spectrum, such as clindamycin and vancomycin, are advised for patients who are allergic to *β*-lactam antibiotics (34).

According to our knowledge, this is the first study to demonstrate that postoperative antibiotic treatment is a protective factor for breast implant infections. In addition, this is the first study to independently identify risk factors for postoperative infection, through robust quantitative meta-analysis. Notably, we included the most recent literature to elaborate and support our results. Moreover, our study compensates for the deficiencies of the previous meta-analysis (RR: 0.80, 95%CI, 0.60–1.07), which did not clarify the protective effect of postoperative antibiotics against infection after breast implantation. In terms of antibiotic duration of action, administering antibiotics until drainage removal after breast implant reconstruction is obviously useful in controlling infection (RR: 0.49, 95% CI, 0.30–0.82).

However, our article still has some limitations. Firstly, our review was confined to English-language literature, which may result in linguistic bias. Besides, possible publication bias, such as the under-publishing of unfavorable statistical results, may have an impact on the findings and should not be overlooked. Secondly, we identified significant variability in our research study. Although we conducted several subgroup analyses, including geographic region, sample size, publication years, duration of postoperative antibiotic use, quality assessment and confounders controlled, the objective heterogeneity observed cannot be ignored. Thirdly, in the included studies, data sources were only provided by a single institution, and sample size was small in most of the studies. To enhance the confidence of the findings of the study, future studies should be performed on a large scale and on a multicenter clinical verification-manner. Moreover, antibiotic regimens are not uniform, including the type of antibiotic, duration of administration and dose. The choice of antibiotic regimen may vary from one clinician to another, which makes it impossible to ensure the robustness of the study. Fifthly, prior to the administration of antibiotics, physicians were neither given indicators of infection nor the results of bacterial cultures, and treated patients based on experience alone, which negatively impacted the results of the study. Last but not least, we focused on the antibiotic regimen in the initial data extraction, but due to the lack of data, we only analyzed the association between antibiotic duration and infection and were unable to perform a statistical analysis of the relationship between the specific antibiotic types and infection, which was also a deficiency of this study.

The results of this study provide scientific evidence that postoperative antibiotics significantly reduce the incidence of infection after breast implantation (RR: 0.65, 95% CI, 0.46–0.90). However, there is a lack of universal consensus in the plastic surgery literature regarding the optimal timing and duration of antibiotic regiments after breast reconstruction (34). In the future, more high-quality multi-center randomized controlled studies are essential. In the meantime, surgeons should concentrate on more detailed protocols for antibiotic application on the present foundation. From our perspective, researchers should also focus on the stratification of infections or other related complications, which would lead to a more precise utilization of our medical resources. More standardized and universal definitions of infection and antibiotic regimens are also required, which will require a concerted effort by the scientific and medical community.

## Data Availability

The original contributions presented in the study are included in the article/Supplementary Material, further inquiries can be directed to the corresponding author/s.
